# Repurposing Ivermectin for COVID-19: Molecular Aspects and Therapeutic Possibilities

**DOI:** 10.3389/fimmu.2021.663586

**Published:** 2021-03-30

**Authors:** Zena Wehbe, Maya Wehbe, Rabah Iratni, Gianfranco Pintus, Hassan Zaraket, Hadi M. Yassine, Ali H. Eid

**Affiliations:** ^1^ Department of Biology, Faculty of Arts and Sciences, American University of Beirut, Beirut, Lebanon; ^2^ Department of Internal Medicine, Basingstoke & North Hampshire Hospital, Basingstoke, United Kingdom; ^3^ Department of Biology, College of Science, United Arab Emirates University, Al-Ain, United Arab Emirates; ^4^ Department of Medical Laboratory Sciences, College of Health Sciences, and Sharjah Institute for Medical Research, University of Sharjah, Sharjah, United Arab Emirates; ^5^ Department of Biomedical Sciences, University of Sassari, Sassari, Italy; ^6^ Department of Experimental Pathology, Immunology and Microbiology, Faculty of Medicine, American University of Beirut, Beirut, Lebanon; ^7^ Center for Infectious Disease Research (CIDR), Faculty of Medicine, American University of Beirut, Beirut, Lebanon; ^8^ Biomedical Research Center, Q.U. Health, Qatar University, Doha, Qatar; ^9^ Department of Basic Medical Sciences, College of Medicine, Q.U. Health. Qatar University, Doha, Qatar; ^10^ Biomedical and Pharmaceutical Research Unit, Q.U. Health, Qatar University, Doha, Qatar

**Keywords:** COVID-19, SARS-CoV-2, ivermectin, coronavirus, mechanism of action

## Abstract

As of January 2021, SARS-CoV-2 has killed over 2 million individuals across the world. As such, there is an urgent need for vaccines and therapeutics to reduce the burden of COVID-19. Several vaccines, including mRNA, vector-based vaccines, and inactivated vaccines, have been approved for emergency use in various countries. However, the slow roll-out of vaccines and insufficient global supply remains a challenge to turn the tide of the pandemic. Moreover, vaccines are important tools for preventing the disease but therapeutic tools to treat patients are also needed. As such, since the beginning of the pandemic, repurposed FDA-approved drugs have been sought as potential therapeutic options for COVID-19 due to their known safety profiles and potential anti-viral effects. One of these drugs is ivermectin (IVM), an antiparasitic drug created in the 1970s. IVM later exerted antiviral activity against various viruses including SARS-CoV-2. In this review, we delineate the story of how this antiparasitic drug was eventually identified as a potential treatment option for COVID-19. We review SARS-CoV-2 lifecycle, the role of the nucleocapsid protein, the turning points in past research that provided initial ‘hints’ for IVM’s antiviral activity and its molecular mechanism of action- and finally, we culminate with the current clinical findings.

## Introduction

SARS-CoV-2 is a positive-sense RNA β-coronavirus, enclosing a capped polyadenylated 30 kb genome, which is the largest among RNA viruses (1). SARS-CoV-2 binds to the ACE2 enzyme on the surface of the target host cell by way of its outer spike protein (S) (2). The receptor-binding domain (RBD) on the S1 subunit interacts with the peptidase domain of ACE2. After partitioning into the host membrane, sequential enzymatic cleavages ultimately lead to the release of the viral genome into the cell (3).

The development of successful vaccines has been a priority in the pharmaceutical and scientific community (4). However, the time between the initial SARS-CoV-2 outbreak in December 2019 until the pharmaceutical companies began vaccine distribution spanned over a year (5). During this period, two million people have died worldwide, according to the World Health Organization (WHO). Moreover, the increasing mutations detected in the S protein have raised concerns that virus evolution might outpace vaccine rollout and the time needed to reach herd immunity (6, 7). Additionally, while vaccines are the main stay for halting the pandemic, it remains critical to develop therapeutics to treat patients and reduce the disease burden.

The drug ivermectin (IVM) has recently been shown to inhibit replication of SARS-CoV-2 in cell cultures (8). IVM is a widely used drug, known best for its antiparasitic properties in both veterinary and human medicine. It was first discovered in the 1970s by microbiologist Satoshi Omura and parasitologist William Campbell (9). Fifty years later, this same drug is suddenly at the forefront of the race against the current pandemic, namely *via* its unintentional inhibition of nuclear transport. It is important to understand and elucidate the ‘journey’ of how IVM emerged as a therapeutic agent against SARS-CoV-2, to follow this precedent and encourage repurposing available drugs for an increasing number of diseases. As such, we aim to highlight essential steps and components in the SARS-CoV-2 lifecycle, the significance of the nucleocapsid protein, the anecdotal evidence that hinted its potential as an anti-viral drug and its molecular mechanism of action. Finally, we summarize real-time results of current clinical trials.

## SARS-CoV-2 Lifecycle

### Initial Formation of the Replicase-Transcriptase Complexes

The basis of the seemingly successful repurposing of IVM is rooted in the identification of important components encoded by the viral genome. The SARS-CoV-2 viral genome encodes non-structural, structural, and accessory proteins. Its positive mRNA strand is translated within the host cell in order to, first, produce its own replication machinery, and second, to produce the structural components required to house viral progeny (10). Two-thirds of the genome code for two large polyproteins, pp1a and pp1ab. Once formed, the polyproteins are subsequently cleaved into 16 individual non-structural proteins (nsps), which primarily provide enzymatic activity (11). Three nsps (1–3) are cleaved by papain-like proteases (PLpro), which itself is localized within nsp3, and the rest are cleaved by the main protease (3C-like protease, 3CLpro) on nsp5 (1). As such, translation of the viral PLpro and 3CLpro are essential for efficient reproduction of the virus. Once the nsps are available, they cooperatively form the replicase-transcriptase complexes (RTCs), which are required for the production of new virions (12). Some nsps (3,4 and 6) induce the development of double membranes from the endoplasmic reticulum (E.R.), Golgi apparatus (G.A.) or the ER-Golgi intermediate compartment (ERGIC), which serve as foci for viral genesis (12). Collectively, the rest of the nsps in the RTC include RNA polymerase, helicase, exoribonuclease, and methyltransferase, among many others. The exact mechanism of replicating its own genome is still under investigation. However, it is understood that negative-sense intermediates are initially formed and then serve as templates for reproducing both genomic and sub-genomic positive-sense RNAs (13). A potential model for the RNA replication in SARS-CoV-2 has been postulated and it is based on homologous proteins in SARS-CoV-1 (10).

### The Importance of the Nucleocapsid Protein

Structural proteins are highly conserved among the various genera of coronaviruses. They include the spike protein (S), the envelope protein (E), the nucleocapsid protein (N) and the membrane protein (M). Once the structural proteins are synthesized, and the viral RNA is reproduced, the S, M and E become embedded within the previously formed double membranes from the host E.R. and eventually reach ERGIC. Meanwhile, the N protein which is tethered to the newly formed genome ‘delivers’ this RNA into S-M-E-embedded ERGIC membrane, leading to the formation of ‘pockets’ which eventually seal off into new virions (1). The interaction of N with the 3’-end of the viral genome is mediated *via* nsp3 (14), the largest subunit of the RTC. The nsp3 acidic ubiquitin-like N terminal domain (UbI1) binds to a serine- and arginine-rich domain in the N protein, thereby anchoring the viral genome to the RTC in order to facilitate RNA replication and, importantly, to eventually ensure the localization of the newly synthesized genome within the viral envelope (Hurst, Koetzner, & Masters, 2013). Ultimately, the N protein is incorporated in the RNA helical structure, which underlies the envelope (15). Overall, the N protein enhances coronavirus transcription, interacts with the viral genome and with M in the viral envelope. Notably, inhibition of N was shown to greatly suppress viral replication, suggesting it is an essential factor in efficient virion production (14, 15). Interestingly, N is the highest expressed protein in infected cells, further corroborating its importance in the viral life cycle (15).

## The SARS-CoV-2 Nucleocapsid Protein Enters the Nucleus

### The Role of Importins

Although RNA replication and translation occur in the cytosol, nuclear access is a key event in the infectious cycle of several viruses, including coronaviruses (1, 8). However, the entry of proteins into the nucleus is a tightly regulated process. To evade this limiting barrier, some viral proteins exploit the importin (IMP) superfamily of nuclear transporters to gain nuclear access (16). Nucleocytoplasmic trafficking is mediated *via* transmembrane nuclear pore complexes (NPCs) in the nucleus, composed of nucleoporin (NPR) subunits. A major class of NPRs known as FG-NPRs are distributed throughout the NPCs and enable nucleocytoplasmic transport due to their interaction with IMP transporters (17). The major IMP classes include IMPα and IMPβ. Nuclear import is mainly mediated either by IMPβs or by heterodimers of IMPα/IMPβ1 (17–19). For cytosolic protein cargo destined for nuclear import, IMPs, particularly IMPα proteins, recognize nuclear localization signals (NLS) on target cargo proteins, whereas IMPβ facilitates the actual transport *via* the NPCs (18). Efficient target binding to the IMPs/EXPs is further supported by Ran, the small monomeric GTPase (20). Active Ran causes dissociation of IMPβ from the importin/NLS-protein complex, releasing its tethered cargo into the nucleus (21). Thus, for a potential SARS-CoV-2 protein to reach the nucleus, it must contain an NLS, properly interact with IMP proteins and Ran must be activated.

### SARS-CoV-2 Nucleocapsid Protein Contains an Enhanced Nuclear Localization Signal

As it happens, the SARS-CoV-2 N contains NLS motifs. Of great significance is the finding that NLS regions in the N gene of SARS-CoV viruses are highly variable compared to the NLS of other coronavirus clades (22). Importantly, these changes occurred during the recent evolution of the highly pathogenic coronavirus clades- including SARS-CoV-2 (22). Incidentally, the numerous nucleotide insertions and deletions within the NLS are associated with enhanced nuclear translocation. Three NLS motifs have been detected on the N of SARS-CoV-2, SARS-CoV, MERS-CoV and seasonal coronaviruses. Uniquely, as a result of the nucleotide variations found in SARS-CoV-2 and SARS-CoV-1, all three NLS motifs contain a distinctly higher overall positive charge among the peptides compared to the less virulent coronaviruses. The higher positive charge of NLS renders the entire N protein also more positively charged and subsequently enhances its efficacy (23). It has been previously corroborated in animal studies that the enhanced translocation of viral Ns to the nucleus results in more severe pathogenicity (24). Therefore, it is possible that these more positively charged Ns, which are characteristic of SARS-CoV-2, may be partially responsible for the associated detrimental effects.

### The Putative Role of the Nucleocapsid Protein Within the Nucleus

It was previously shown that viral proteins that enter the nucleus might suppress host genes related to the anti-viral response, leading ultimately to increased pathogenicity (25). This may also be the case with SARS-CoV-2, as *in vitro* studies indicated that the SARS-CoV-2 NP could interact with dsDNA, possibly due to its high positive charge and the negative charge of DNA (26). Although the exact activity of the SARS-CoV-2 N within the nucleus has not been fully characterized, previous examination of several coronavirus Ns can offer insight (24).

The N of the coronavirus infectious bronchitis virus (IBV) was detected not only in the cytoplasm but also within the nucleolus. Nucleolus targeting was also shown with the SARS-CoV-1 N (27). It is important to note that the presence of N in the nucleus was indispensable for the replication of IBV, highlighting that cytosolic activity was not sufficient. In another related coronavirus, mouse hepatitis virus (MHV), nuclear proteins were also implicated in its replication. MHV N was specifically detected in the nucleolus, which itself is formed during interphase of the cell cycle and allows formation of ribosomal RNA (rRNA) and ribosomal subunits. The reason for N targeting of the nucleolus is not entirely understood. However, it is possible that N associates with rRNAs, in order to ‘reserve’ their use for translation of sub-genomic RNA. It was also shown *in vitro* that N transfection into cells resulted in multi-nucleate cells, indicating the delay of cytokinesis (24). This would provide favorable and prolonged conditions for the virus intracellularly to continue to synthesize its genome and sub-genome, translate its proteins and enable sufficient virion packaging. Moreover, N is proposed to dampen the host cell’s antiviral transcriptional response within the nucleus (8). Nevertheless, confirming the presence SARS-CoV-2 N in the nucleolus and understanding its role would elucidate the pathogenicity of this virus.

**Figure 1:**
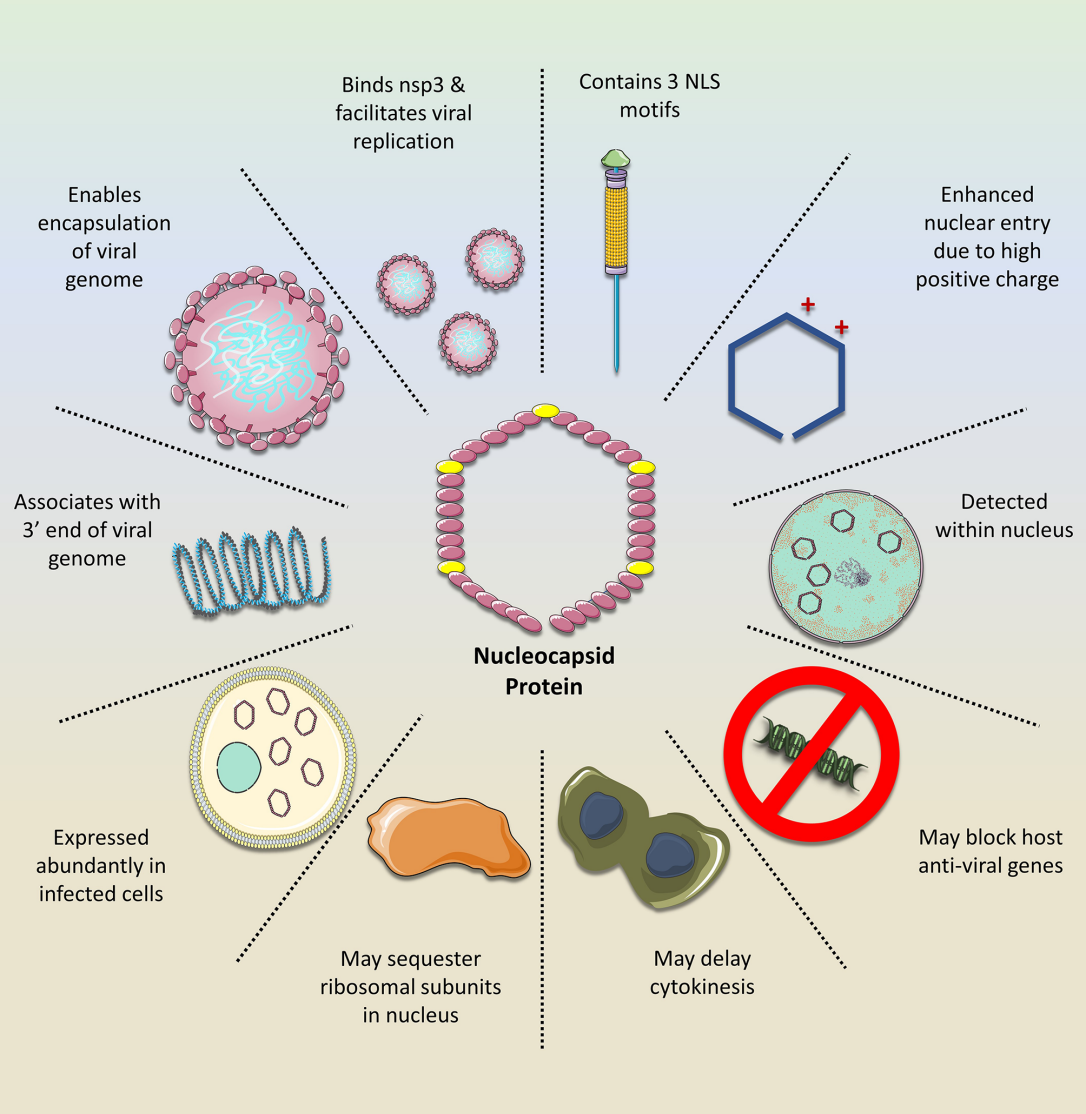
The importance of the SARS-CoV-2 nucleocapsid protein (N). The N exerts numerous functions that facilitate viral replication while mitigating the host cell response. Owing to its NLS motifs, the protein retains a relatively high positive charge, compared to the N of other coronavirus clades. This enhances its transport into the nucleus where it may silence host anti-viral genes while sequestering ribosomal subunits, possibly for viral mRNA translation, as demonstrated with the N of other related viruses. Moreover, the N is important for stabilizing the interaction between the viral mRNA and nsp3 protein, which facilitates genome replication. In addition, it tethers the newly emerged viral RNA to the viral envelope, ultimately allowing for its encapsulation and formation of new viral progeny. Given these features and its abundance in the infected cell, it would be a promising drug target against SARS-CoV-2.

N is an essential component of newly formed virions as it ensures a proper ‘delivery’ of the replicated viral RNA genome within the developing envelope (28, 29). Moreover, it is essential for proper viral RNA dependent RNA polymerase activity, as demonstrated in Influenza A (29). As such, targeting the activity of N would offer a potent antiviral activity against SARS-CoV-2. In fact, N was shown to be an effective anti-viral target against Influenza A. One of the useful properties of N is its numerous binding sites, which have been shown to accommodate various drugs (29, 30). For example, compounds which can target the tail-loop binding pocket abrogate N oligomerization, while the compound F66 binds to the RNA-binding groove of the protein and is associated with improved survival in animal models infected with Influenza A (29). **Figure 1** illustrates how the N of SARS-CoV-2 facilitates virus replication and mitigates the host cell response, thus further strengthening its position as a promising target of anti-viral drugs.

## Ivermectin

### The Discovery of Ivermectin

IVM was originally discovered from organisms that were isolated from soil samples collected from the woods nearby to Kitasato Institute in Kawana, Japan. Fermentation products released by a bacterium from the soil, which was later classified as *Streptomyces acermitilis*, appeared to exhibit antiparasitic activity (specifically against *Nematospiroides dubius*). Purification and isolation of the bioactive compounds showed naturally occurring macrocyclic lactones, and these were subsequently named avermectins. Avermectins are made up of four compounds, which exist as two variants: A_1_, A_2_, B_1_, and B_2_. Variants ‘A’ and ‘B’ indicate the presence of methoxy or hydroxyl groups, respectively, at the C5 position. Number ‘1’ describes the double bond between C_22_ and C_23_. On the other hand, number ‘2’ indicates the presence of hydrogen at C_22_ and a hydroxyl group at C_23_. B_1_ avermectins were proven to be most active on oral administration, and on this basis, IVM was chemically derived. IVM contains an 80:20 combination of 22,23-dihydro-acvermectin B_1a_ and 22,23-dihydro-avermectin B_1b._ Its antiparasitic effects are primarily caused by high-affinity irreversible binding to glutamate-gated chloride (Cl^-^) channels located on nerve and muscle cells of nematode, which leads to hyperpolarization (9, 31). Ultimately, the increased permeability to Cl^-^ results in paralysis and death of the nematode (31).

As of yet, IVM has treated hundreds of millions of people with onchocerciasis, most commonly given at 150-200 μg/kg of body weight for one dose initially, and repeated at 6-12 monthly intervals as appropriate (32). Its use extends to a broad spectrum of parasitic nematodes on both oral and parenteral administration, and is also effective against arthropods, including lice (33).

Importantly, IVM was approved by the FDA for human use in 1987 (34). Its low toxicity and safety are attributed to the fact that its human target receptors are ‘secluded’ in the CNS, and IVM has not been shown to cross the blood-brain barrier. In addition, IVM displays a 100-fold greater affinity for parasitic Cl^-^ channels compared to the human homologs (35). Moreover, severe detrimental effects in humans were shown only in those who over-dosed using approximately 15.4 mg/kg body weight IVM, which is 77 times above the prescribed dose. This corroborates the advantage of repurposing drugs, as these medications have already been tested arduously and extensively to confirm their efficacy and safety, thereby decreasing the transit time from shelf to intake.

### Screening for Inhibitors of Nuclear Import

The potential of IVM as an inhibitor of nuclear transport of viral proteins was initially suggested in 2011. Initially, Wagstaff and colleagues screened for nuclear import inhibitors, which block the interaction between IMPs and potential target cellular proteins (21). They randomly selected 480 compounds from LOPAC^1280^ (Library of Pharmacologically Active Compounds; Sigma, St. Louis, MO). IVM surfaced as a drug that generally inhibits IMP activity (21). A year later, they confirmed that this apparent activity of IVM also inhibits nuclear transport of viral proteins HIV and Dengue virus in HeLa cells (36). Specifically, it was shown that GFP-tagged IMP was significantly reduced in the nucleus of HeLa cells after 3 hrs. of co-incubation with IVM (36). Moreover, the effect was unique to IMPα/β interactions and did not affect proteins bound only to IMPβ1. The importance of blocking the nuclear import of viral proteins emerged when it was later shown that IVM also prevented replication of HIV (36). As such, it surfaced as a possible repurposed drug, capable of preventing viral cargo from interacting with IMPα/β for nuclear import, with the potential to result in viral ‘death’ (21, 36).

Soon after, the effect of IVM against the nuclear import of viral proteins was further validated. For example, IVM prevented nuclear translocation of nsp5 in Dengue virus, West Nile virus, and influenza and inhibited transport of large tumor antigen (T-ag) in simian virus (25, 37, 38). The Wagstaff et al., 2011 and 2012 studies were pivotal in providing much of the initial rationale for the recent consideration of IVM as a SARS-CoV-2 antiviral agent. In fact, it was the same researchers who nine years later, in 2020, demonstrated that IVM inhibits SARS-CoV-2 *in vitro* replication (8).

Given its efficacy in inhibiting nuclear import of other viral proteins, the anti-viral effect of IVM against SARS-CoV-2 was evaluated shortly after the pandemic erupted (8). Specifically, Vero/hSLAM cells were inoculated with SARS-CoV-2 isolate for 2 hrs., followed up with supplementation of 5 μM of IVM. Within 24 hrs. after treatment, there was a 93% reduction of viral RNA in the supernatant and 99.8% reduction of cellular viral RNA, compared to controls. After 48 hrs., there was a further 5000-fold reduction of viral RNA in the supernatant as well as the cell pellets, indicating that cells were essentially ‘cleared’ of SARS-CoV-2 (8). Although IVM possessed a potent antiviral activity (IC_50_= ~2μM), no cytotoxicity was detected at any time points in this study (8).

### IVM Specifically Interacts With IMPα

**Figure 2:**
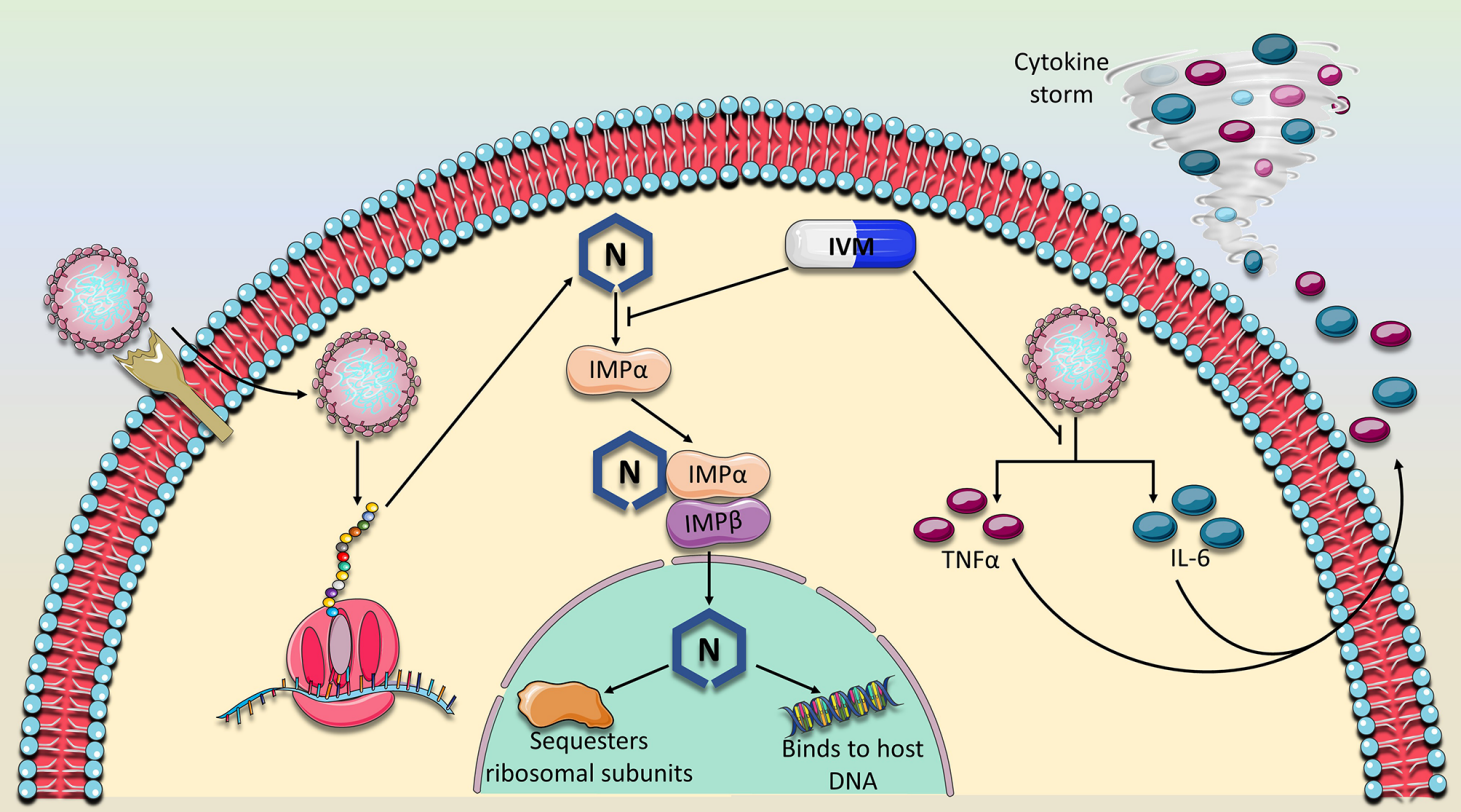
Proposed mechanism of action of Ivermectin against SARS-CoV-2. IVM has previously been established as a nuclear import inhibitor by binding to and antagonizing the ability of the importin (IMPα) to bind to its target cargo. Because the nucleocapsid (N) protein contains a nuclear localization signal, IVM is expected to prevent the binding of IMPα to the N binding site. Consequently, N would not perform its nuclear activity which is thought to suppress the host immune response and sequester ribosomal subunits, mechanisms which are thought to abrogate sufficient viral replication. In addition, the expression of two major cytokines, TNFα and IL-6 which drive the detrimental cytokine storm in COVID-19 patients were also shown to be dampened in the presence of IVM. As of yet, these two major mechanisms which involve viral replication and immune response suppression appear to characterize the main activities of IVM against SARS-CoV-2.

IVM was shown to specifically inhibit IMP α/β mediated nuclear import required for replication of HIV-1 and Dengue virus, and therefore it was proposed as the potential mechanism by which it inhibits SARS-CoV-2 (36). Indeed, this baton was passed on, and a subsequent study verified the IVM-IMPα interaction in host cells (38). In fact, it was shown that IVM not only inhibits IMPα association with IMPβ, but can even dissociate IMPα/β heterodimers (38). The specific binding target of IVM was identified, using CD spectroscopy, to be the alpha-helical rich ‘armadillo’ (ARM) domain of IMPα. Moreover, as concentrations of IVM increased, alpha helices in the ARM domain became increasingly destabilized. No changes were detected in the structure of IMPβ. They further verified that this observed effect on IMPα impaired its binding to NLS-containing nsp5 from Dengue Virus (38). As such, preventing N interaction with IMPα, is a likely mechanism that contributes to IVM’s ability to hinder SARS-CoV-2 *in vitro* replication (**Figure 2**).

#### The Implications of Disrupting IMPα Activity for the Host Cells

Because IVM emerged as a general inhibitor of IMPα-dependent nuclear cargo, it is important to consider the implications this may have on host cell proteins and functions. However, any effect would likely be non-detrimental given the safety record of IVM over the past 50 years and its transient prescription for an acute disease (31).

Notably, expression levels of IMPα vary in a cell and developmental specific manner, particularly during differentiation processes (39). Animal knock-out studies for *impα*, highlighted its essential role in reproductive organ development. Specifically, *impa-/-* mice developed lower reproductive organ function in females, including insufficient follicles’ growth during the maturation stage in the ovaries, incomplete uterus construction, and reduced serum progesterone (40). Moreover, estrogen-responsive genes were also not efficiently expressed, indicating IMPα may be involved in hormonal regulation. Other cells like muscle stem cells underwent apoptosis and depletion (39).

IVM was also shown to disrupt the oxygen regulatory mechanisms (41). Hypoxia-induced transcription factors (HIFs) regulate cellular adaptation to decreased oxygenation within the cell. Hypoxia renders the HIF subunit, HIFα stable and causes it to accumulate within the nucleus where it induces transcription of genes that may readjust oxygen levels. HIFα translocation into the nucleus requires nuclear import in an NLS-IMPα/β dependent manner. Indeed, it was shown that IVM results in decreased association between HIFα and IMPα, preventing its path into the nucleus. Subsequently, nuclear HIFα and transcription of target oxygen-regulatory genes was reduced (41).

Pharmacokinetic studies conducted by MERCK show that IVM plasma concentrations peak after 4 hours, following 12 mg doses in healthy human volunteers (42). Subsequently, it is metabolized in the liver and its break down products are mainly excreted in the feces over a period of 12 days. Its half-life is around 18 hrs. Moreover, it was shown that it does not bind permanently to its target proteins.

### Other Possible Modes of Antiviral Activity by IVM

A recent molecular docking study demonstrated that in addition to IMPα, IVM showed high binding affinity to the viral RNA-dependent RNA polymerase (RdRp) complexed with RNA helicase compared to other 10 viral targets included in the analysis (43). However, it was later shown that IVM does not bind to viral RdRp in both Zika virus (Z.V.) and Dengue virus (38). It remains to be identified if IVM may bind to RdRp in coronaviruses.

Other mechanisms of IVM action have also been identified (**Figure 2**). For example, it previously was shown to suppress the production of Interleukin-6 (IL-6) and Tumor Necrosis Factor alpha (TNFα), two major components of the detrimental cytokine storm induced by SARS-CoV-2 (44). Moreover, a study in Syrian hamsters showed that IVM did not affect SARS-CoV-2 viral load but overall dramatically reduced IL-6/IL-10 ratio and modulated infection outcomes (45). Specifically, hamsters that were inoculated with SARS-CoV-2 were subcutaneously injected with IVM (0.4 mg/kg body weight). IVM reduced severity of clinical symptoms in males, but completely eliminated symptoms in females, which suggests a gender-specific effect of this drug and a factor that should be considered in clinical trials. The gender-specific modulation of IVM on cytokines was also apparent. While females displayed lower levels of cytokines such as IL-6, INFγ and TNFα, males on the other hand developed an enhanced production of INFγ. Notably, viral load in nasal and lung tissues, as well as viral replication rate were not altered in either gender after administration of IVM (45). This is in contrast to the finding that IVM significantly blocks viral replication *in vitro* and it may be attributed to the much higher dose of IVM that was used (8). However, it is important to note that the dose of IVM that was used on the cells (5 μM) is approximately 50-fold higher than the normal C_max_ associated with one dose of IVM (200 μg/kg) (46, 47). Therefore, it is important to establish a dose-dependent effect of IVM on viral load and safety in human COVID-19 patients at various doses.

Further, IVM was shown to induce an elevated level of IL-6 and TNFα in onchocerciasis patients, two days after a single dose (150 μg/kg body weight) (48). However, this was attributed to the destruction of the parasite microfilariae, which would usually not be a factor in COVID-19 patients.

Thus far, studies on IVM highlight that it remains important to identify the specific dose of IVM that may reduce viral load, without adverse effects, in humans and to understand if it will differentially affect male and female COVID-19 patients.

### Adverse Effects Reported in Animals and Humans in Previous Studies Using Ivermectin

The direct toxic effects of IVM were first identified in animal studies, mainly as an antiparasitic treatment. The vast amount of evidence around the use of IVM exists using dose regimens of 150-200 μg/kg of body weight. Hence, the risks and associated side effects are mostly reported at these doses. Studies suggest the common adverse effects are rash, headache, nausea and dizziness, while transient tachycardia is rare and self-limiting (49). Other effects include ataxia, sweating, tremors, and in some cases, coma and death (50).

A retrospective study looking at residents of an extended care facility showed increased rates of death in patients treated with IVM for resistant scabies. These study results were criticized due to some significant limitations of the study, and therefore, the deaths of these residents could not be reliably attributed to the IVM. For example, there was no control of the lasting previous drugs used to treat the scabies, some of which are known for their toxic effects. Importantly, IVM’s toxic effects are short term and are usually resolved (51).

Studies exploring the adverse event profiles of patients on high doses of IVM have also been conducted. Higher dose levels (300-1000 μg/kg) were administered to healthy individuals with head lice, as part of a double-blind and randomized trial, with adverse events reported as having no clinical or biochemical significance (50).

## Clinical Trials

### The Effect of Ivermectin on SARS-CoV-2 Patients

Soon after IVM emerged as a potential therapeutic agent, clinical trials on COVID-19 patients ensued. However, the available published data and ongoing clinical trials, which are summarized in **Table 1**, do not provide a clear and uniform understanding of the effect of IVM on COVID-19 patients. This is mainly due to small sample sizes (n=12-203) and the lack of information specifying when exactly IVM is administered after testing positive for SARS-CoV-2 (46, 52–54). It is important to highlight how soon after testing positive the patient receives IVM, in addition to the degree of COVID-19 severity, in order to understand if the effect of the drug is dependent on time and symptom severity. Additionally, several studies are retrospective in which investigators examined past COVID-19 patients who were prescribed IVM, without proper placebo control groups (46, 53). Moreover, most of the studies utilize the antiparasitic effective dose for IVM (0.2 mg/kg body weight), which is substantially less than the equivalent *in vitro* dose of IVM used against SARS-CoV-2 (8, 53, 54). Nevertheless, the available data does indicate that IVM may, in fact, be effective against COVID-19.

**Table 1:** Outcomes in Current Clinical Trials at Ivermectin.

Type of study	Treatment Groups	Adverse Events due to IVM		Significant decrease inBlood Biomarkers or Clinical Symptoms		Significant decrease in viral clearance or viral load	Significant decrease in Mortality and/or ICU transfer
**(** 52 **)** • **Randomized, Placebo-controlled trial** • **Mild COVID-19 (fever, cough, or sore throat)** • **No underlying comorbidities**	**Group 1:** IVM (12 mg for 5 days) **n = 24**	None Reported		**↓ CRP (P<0.02), day 5.** N.S* in clinical symptoms by day 7	**9.7 days viral clearance significantly reduced day 7 & 14 compared to placebo (p<0.03)**		N/A
	**Group 2**: IVM (12 mg once) + doxycycline (200 mg on day 1, 100 mg every 12 h for the next 4 days). **n = 24**	None Reported		N.S in clinical symptoms by day 7	11.5 days viral clearance (N.S with Group 3)		N/A
	**Group 3:** Placebo control group, **n=24**	None Reported		No N.S in clinical symptoms by day 7	12.7 viral clearance (N.S with Group 2)		N/A
**(** 53 **)** • **Retrospective study of** **hospitalized patients with confirmed SARS-CoV-2 during their admission, who had received IVM.** • **Included patients older than 18 and with underlying morbidities.**	**Group 1:** IVM (200 μg/kg B.W and those who had received a 2nd dose at day 7) + hydroxychloroquine and/or azithromycin. **n = 173**	None Reported		N/A	7 days (N.S) viral clearance		**15% of patients died (P=0.03)** For the subset of patients receiving oxygen support **38.8% died (p=0.001)**
	**Group 2**: Only provided standard care. **n = 107**	None Reported		N/A	7 days (N.S) viral clearance		25.2% of patients died For the subset of patients receiving **oxygen support 80.7% died**
**(** 46 **)** • **Retrospective study of 26 previously hospitalized severe COVID-19 patients, who had received IVM. Patients were compared to those who did not receive IVM treatment.**	**Group 1:** IVM (200 μg/kg, single dose after 12 days since onset of symptoms**) +** Immunosuppressant drugs (tocilizumab, corticosteroids and/or anakinra), treated with **n = 13**	None Reported		N.S in either	N/A		N/A
	**Group 2:** Immunosuppressant drugs (tocilizumab, corticosteroids and/or anakinra) **n = 13**	None Reported		N.S in either	N/A		N/A
**(** 54 **)** • **Randomized, double-blind, placebo-controlled trial using recently diagnosed patients with mild SARS-CoV-2 and 72 hrs. within onset of fever or cough** • **Excluded patients with risk factors for complicated disease**	**Group 1:** IVM (400 μg/kg Body weight) **n = 12**	None		N.S in blood biomarkers **50% reduced** **hyposmia/anosmia. (P<0.001)**	N.S		N/A
	**Group 2:** placebo **n = 12**	None		N.S in either	N.S		N/A
**Ongoing Clinical Trial # NCT04422561** • **Randomized, controlled trial for IVM as a prophylactic treatment for asymptomatic family members of new COVID-19 patients.**	**Group 1:** IVM (Ivermectin Tablets: 40-60 kg (15mg/day) 60-80kg (18mg/day) >80kg (24mg/day), 2 doses 72 hours apart)) **n = 203**	**5% reported adverse events (gastro-intestinal G.I fatigue, numbness) compared to 0% control** ***(statistical significance has not been calculated)***		N/A	N/A		0% mortality
	**Group 2**: no IVM **n = 101**			N/A	N/A		0% mortality
**Ongoing Clinical Trial # NCT04343092** • **Interventional study, single group assignment to test effect of IVM on hospitalized COVID-19 patients with pneumonia. Experimental group was compared to historical control population.**	**Group 1:** Ivermectin (0.2 mg/kg body weight) + Hydroxychloroquine 400 mg at admission day then 200 mg for 5 days + azithromycin 500mg at admission day then 250 mg for 5 days **n=16**	**0% adverse events related** **to IVM *(statistical significance not indicated)***	N/A		N/A		**0% mortality *(statistical significance not indicated)***
	**Group 2:** Historical group of patients who did not receive IVM, only Hydroxychloroquine 400mg at admission day then 200 mg for 5 days + azithromycin 500mg at admission day then 250 mg for 5 days **n = 16**	** **		** **			**N/A**
**NCT04523831** • **Interventional randomized placebo-controlled study to** **evaluate the effect of IVM in hospitalized and non-hospitalized patients with mild to moderate COVID-19 infection**	**Group 1:** Ivermectin 12 mg + Doxycycline 100 mg twice daily for 5 days and Paracetamol, Vitamin D, Oxygen if indicated, Low molecular weight heparin, dexamethasone if indicated **n = 183**	**1.09% serious adverse effect (erosive esophagitis)** **3% Adverse Effect (non-ulcer dyspepsia)**					**0% mortality** **(significance is not indicated)**
	**Group 2:** Paracetamol, Vitamin D, Oxygen if indicated, Low molecular weight heparin, dexamethasone if indicated **n = 180**	**0%**					**1.67% mortality** **(significance is not indicated)**

n, number of subjects.

One of the first published studies involved a randomized, controlled double-blind study on 72 hospitalized COVID-19 with mild symptoms (52). Patients that were admitted to the hospital within the last 7 days were either treated with IVM alone (12 mg for 5 days), IVM and doxycycline (12 mg for 1 day, 200 mg doxycycline on day 1 and 100 mg doxycycline every 12 hrs. for days 2-6), or with placebo. The most significant effect of IVM was detected for the rate of viral clearance, measured by a negative rRT-PCR on nasopharyngeal swab. Specifically, the 5-day IVM treatment group demonstrated the fastest rate of viral clearance (approximately 10 days; p<0.02), compared to placebo (approximately 13 days). However, there was no significant difference between the groups for symptoms like cough, sore throat and fever and adverse drug effects (52). Limitations for this study include the exclusion of patients with underlying morbidities and lack of follow-up for mortality and ICU transfers after day 7.

Another recently published clinical study in Florida involved 280 hospital-admitted patients who developed COVID-19 during admission (53). However, it was based on a retrospective analysis of patients and therefore lacked adequate controls. Patients were grouped according to whether or not they received a single dose of IVM (200 μg/kg body weight) and standard care (hydroxychloroquine and azithromycin, unspecified dose) or those who only received standard care. It was not stated, though, at which day post-PCR testing that patients received the drug. The most prominent effect of IVM was the reduction in mortality in the treatment group (15% mortality, p= 0.03) compared to the control group (25% mortality). Mortality was especially reduced in the severe subgroup of patients receiving oxygen support (38.% mortality in IVM group, compared to 80.7% mortality in non-IVM group; p = 0.001). In fact, hospital stay was similar in both groups, as was the rate of viral clearance. However, data was lacking for clinical symptoms as it was not a main outcome (53). In contrast to the previous study, this trial included patients with comorbidities and no adverse drug events were reported. Although this study is limited because it is retrospective, it suggests that IVM may be beneficial in reducing mortality almost by one half, especially for patients receiving oxygen support. Nevertheless, it remains ambiguous as to how soon after testing positive for SARS-CoV-2, patients received IVM, which would have been an important guideline. The results of the few addition published IVM trials are summarized in **Table 1**.

There are currently around 50 clinical trials taking place registered on *clinicaltrials.gov*, which study the effect of IVM as a prophylactic or therapeutic drug (35). Five studies have completed testing at Phase 1, 2 or 3 and three have posted their real-time results, which are included in **Table 1**. One of the most promising outcomes include 0% mortality in COVID-19 patients who developed pneumonia (National Clinic Trial Number NCT04343092), however there is no report of an adequate control group and its corresponding rate of mortality. Another ongoing trial (NCT04523831) on COVID-19 hospitalized and non-hospitalized patients also demonstrated 0% mortality, in the IVM group, compared to 1.67% mortality in the control group. Like published studies, ongoing clinical trials also do not present thorough outcomes and the significance statistics are still lacking. As such, more trials are needed which include proper placebo control groups, testing of various doses and records of numerous outcomes.

### Ivermectin Compared to Other Anti-SARS-CoV-2 Drugs

In addition to IVM, many clinical trials have been conducted to test drugs for COVID-19, many of which have been concluded (47). Most of these medications did not result in significantly improved outcomes, however a few drugs were associated with slightly beneficial effects.

Remdesivir (RMV) has previously been shown to target the viral RNA dependent RNA polymerase in SARS-CoV-1 (55). In the context of SARS-CoV-2, RMV prescribed once daily for 10 days (200 mg day 1, 100 mg days 9-10), showed a survival benefit by days 15 and 28 in patients who did not require oxygen support (56). On the other hand, a large global study by the WHO did not find any clinical benefit for RMV (57). However, an *in vitro* study suggests a possible synergistic effect of combined RMV and IVM. Specifically, EC_50_ of approximately 2.3 μM IVM and 1.9 μM RMV were shown to disrupt viral cytopathic activity (58). Because RMV is FDA-approved for the treatment of COVID-19, it is warranted to explore its effect in combination with IVM in clinical trials.

Dexamethasone (6 mg for 10 days) decreased mortality only in severe cases requiring oxygen and mechanical ventilation but was ineffective for mild cases and did not result in any adverse effects (59). Interestingly, a clinical trial (NCT04425863) involving a combined therapy of IVM (0.6 mg/mL solution), dexamethasone (4 mg injection), aspirin (250 mg tablets) and enoxaparin (injection) did indicate a favorable outcome. All patients with mild COVID-19 symptoms (n=135) fully recovered and their symptoms did not worsen. Of those who entered the study displaying severe symptoms (n= 31), one patient perished.

The use of convalescent plasma is also not entirely promising as preliminary analysis based on 1873 reported deaths among 10,406 randomized patients, shows no significant difference in the primary endpoint of 28-day mortality (18% convalescent plasma vs. 18% usual care alone, p=0.34). Although some early studies showed some clinical benefits for convalescent plasma in COVID-19 patients (47), a recent press release from the largest randomized clinical trial, known as the ‘RECOVERY Trial’, revealed otherwise (60). The investigators concluded no evidence of benefit for convalescent plasma in treatment of COVID-19, whereby the 28-day mortality did not differ significantly between the treatment and the control groups. Recently two clinical trials showed that monoclonal antibodies against the spike protein can disrupt progression of early COVID-19 infection (61, 62). However, this type of therapeutic remains very expensive and largely unavailable.

Finally, a recent study, still awaiting peer-review, demonstrated that treatment with the IL-6 receptor antagonists, tocilizumab and sarilumab, improved the clinical outcome including survival in critically ill COVID-19 patients (63). However, these drugs remain expensive and not widely available especially in poor and developing countries.

## Concluding Remarks and Perspectives

The available data from IVM clinical trials lack uniformity and have not established the optimal anti-viral dose. However, the evidence does support its safety and efficacy in improving survival rates, especially compared to the other aforementioned drugs. It is important to note that past research has demonstrated the importance of combined, rather than anti-viral monotherapy. Indeed, the use of a single drug does not efficiently suppress long-term replication of the virus (64). As evident by the ongoing clinical trials for the treatment of COVID-19, the most efficient decrease in mortality (0%) was largely a result of multiple prescribed drugs including IVM, hydroxychloroquine and azithromycin or IVM and doxycyline **Table 1**. Given the wide use of numerous drugs to treat COVID-19 patients, it remains imperative to explore the optimal combination of various therapies.

Notably, the clinical outcomes upon prescribing IVM on its own did not result in significantly improved outcomes for COVID-19 patients and nor should it be particularly encouraged (54). In fact, cross-resistance to other medications may be induced as a result of selective pressure resulting from a single medication (64). This may be a likely event as RNA viruses are well noted for their pronounced capacity for mutations, a finding which has already been established also for SARS-CoV-2 (65). Therefore, although IVM may contribute to the suppression of SARS-CoV-2 replication, it is important not to dismiss the risk of selecting for highly pathological and resistant viral strains when using a sole medication. That said, in a recent clinical trial that we have just concluded and is under review, we show that a single dose of IVM can significantly reduce the viral load in asymptomatic SARS-CoV-2 positive subjects. However, in these subjects, zinc and vitamin C were concomitantly used.

The available data thus far suggests a favorable outcome when using IVM in specific doses and in particular drug combinations. It remains imperative to establish the most effective doses, combination, and timing of drug administration as it may largely determine the therapeutic outcome. Although vaccines are currently being distributed, they do not guarantee complete protection against SARS-CoV-2. Therefore, it is important to establish therapeutic alternatives in the event that viral re-infection occurs. Given the promising emerging clinical data from IVM studies and the unprecedented public health threat that the pandemic poses, it is critical that further specific and well-designed studies are carried out to validate the therapeutic potential of IVM.

## Author Contributions

AE generated the concept. ZW and MW wrote the first draft. All authors revised the manuscript and approved it before submission. HY generated funding.

## Funding

This study was supported by Qatar University Grants # QUCG-BRC-20_21 and QUHI-BRC-20/21-1.

## Conflict of Interest

The authors declare that the research was conducted in the absence of any commercial or financial relationships that could be construed as a potential conflict of interest.
